# High precision measurements of the hyperfine structure of Vanadium ions in the ultraviolet range

**DOI:** 10.1038/s41598-026-44426-3

**Published:** 2026-03-16

**Authors:** A. Karadimas, D. Bettaney, P. Campbell, B. Cheal, S. Chinthakayala, R. de Groote, Á. Koszorús, I. Moore, A. Raggio, J. Warbinek

**Affiliations:** 1 Instituut voor Kern- en Stralingsfysica, KU Leuven, Leuven, Belgium; 2 Department of Physics and Astronomy, University of Manchester, Manchester, UK; 3 Department of Physics, University of Jyväskylä, Jyväskylä, Finland; 4 Experimental Physics Department, CERN, Geneva, 1211 Switzerland; 5 Department of Physics, University of Liverpool, Liverpool, UK; 6 SCK CEN, Belgian Nuclear Research Centre, Mol, Belgium; 7 Grand Accélérateur National d’Ions Lourds (GANIL), Caen, France; 8 Université de Caen Normandie, Caen, France

**Keywords:** Chemistry, Physics

## Abstract

High resolution collinear laser spectroscopy has been performed on singly charged ions of $^{50,51}$V at the IGISOL facility of the Accelerator Laboratory, University of Jyväskylä, Finland. Eleven ionic transitions were investigated in the ultraviolet wavelength range, improving the precision of the known hyperfine parameters and providing newly measured values. We report also the isotope shifts between the two natural isotopes for five of the studied transitions. These results provide benchmark data for the electronic structure of vanadium and open the way for future studies of nuclear-structure phenomena along the vanadium isotopic chain using laser spectroscopy techniques.

## Introduction

Vanadium is a transition metal belonging to the group of refractory elements, characterized by high melting points and a remarkable resistance to vaporization^1^. These elements play a crucial role in astrophysics and cosmochemistry, as they are abundantly observed in solar and stellar spectra and serve as tracers of the chemical and nucleosynthetic evolution of the Galaxy^2,3^. With atomic number $Z = 23$, vanadium lies in the iron-group region, where accurate laboratory data on spectroscopic properties are essential for modeling stellar abundances and interpreting high-resolution astrophysical spectra^4^. In particular, vanadium lines are widely used in the analysis of late-type stellar spectra and metal-poor stars, where neglecting hyperfine and isotopic effects can lead to significant errors in deduced elemental abundances^5,6^. Precise laboratory measurements of the hyperfine structure and isotope shifts are therefore indispensable for astrophysical modeling, including the determination of metallicities and the validation of non-LTE (local thermodynamic equilibrium) calculations in stellar atmospheres^6^.

From an atomic-structure perspective, vanadium forms singly charged ions with a ground-state configuration $3d^4$^7^, giving rise to a dense and complex system of fine and hyperfine levels. In the present work, the investigated transitions originate from the low-lying $3d^3 4s$ configuration, which constitutes a metastable state relative to the $3d^4$ ground state. The direct transition between the $3d^4$ ground state and the $3d^3 4s$ configuration is parity forbidden in electric-dipole (E1) order, as both configurations possess even parity. As a consequence, the $3d^3 4s$ levels exhibit long radiative lifetimes and can sustain a significant population in an ion beam environment. The open 3*d* shell and the resulting strong electron-correlation effects make ${V}^{+}$ an excellent benchmark for testing modern relativistic atomic-structure theories, such as multiconfiguration Dirac–Hartree–Fock and configuration-interaction methods, which must accurately describe complex configuration mixing in transition-metal ions^8^. Experimental results of high precision thus provide valuable reference data for the refinement of theoretical approaches and for improving the reliability of atomic models.

From a nuclear-structure point of view, vanadium presents an intriguing case. It has one stable isotope, $^{51}$V, and one extremely long-lived isotope, $^{50}$V, with a half-life exceeding $10^{17}$ years^9^. By measuring nuclear electromagnetic moments and changes in mean-square charge radii of stable and radioactive vanadium isotopes, one can investigate nuclear deformation in the middle of the $f_{7/2}$ proton shell, single-particle character near the magic neutron number $N=28$, isospin symmetry in the self-conjugate nucleus $^{46}$V^10^, and the evolution of nuclear charge radii across the $N=28$ shell closure^11,12^. Although the isotopic chain of vanadium is particularly interesting for exploring nuclear-structure phenomena, so far only $^{50}$V and $^{51}$V have been studied by collinear laser spectroscopy. With spectroscopic information available for only two isotopes, it is not possible to determine the atomic field- and mass-shift factors empirically using King-plot methods^13^, which require at least three stable isotopes with independently known charge radii. Consequently, the extraction of absolute changes in nuclear charge radii from the measured isotope shifts in vanadium requires reliable theoretical input for both the field-shift and mass-shift factors^14,15^.

In this work, we report on high-resolution collinear laser spectroscopy of singly charged vanadium ($^{50,51}$V) ions. Eleven ionic transitions were studied, allowing the extraction of precise magnetic dipole hyperfine constants (*A*) and electric quadrupole hyperfine constants (*B*). The uncertainty of the extracted *A* constants is on the order of $0.1\%$, representing a substantial improvement in precision compared to previous measurements obtained by Fourier Transform Spectroscopy, which are limited in resolution^6^. Isotope shifts between $^{50}$V and $^{51}$V were additionally determined for multiple transitions. Studies in the past have been conducted in atomic vanadium^16–19^ but the results presented here constitute systematic laser spectroscopy studies of vanadium ions and provide benchmark data for the ionic structure of vanadium. They also establish the experimental groundwork for future investigations of short-lived radioactive isotopes of vanadium.

## Experimental methodology

Collinear laser spectroscopy (CLS) is a high-resolution technique in which an accelerated ionic or atomic beam is overlapped collinearly (or anti-collinearly) with a narrow-band laser beam^20^. The well-defined ion-beam velocity enables precise Doppler tuning of the resonance frequency through controlled variation of the acceleration potential, while the collinear geometry minimizes residual Doppler broadening. In combination with ion-beam cooling and bunching, CLS provides high sensitivity and excellent spectral resolution, making it particularly suitable for extracting hyperfine structure parameters and isotope shifts of stable and radioactive isotopes^21^.

**Fig. 1:**
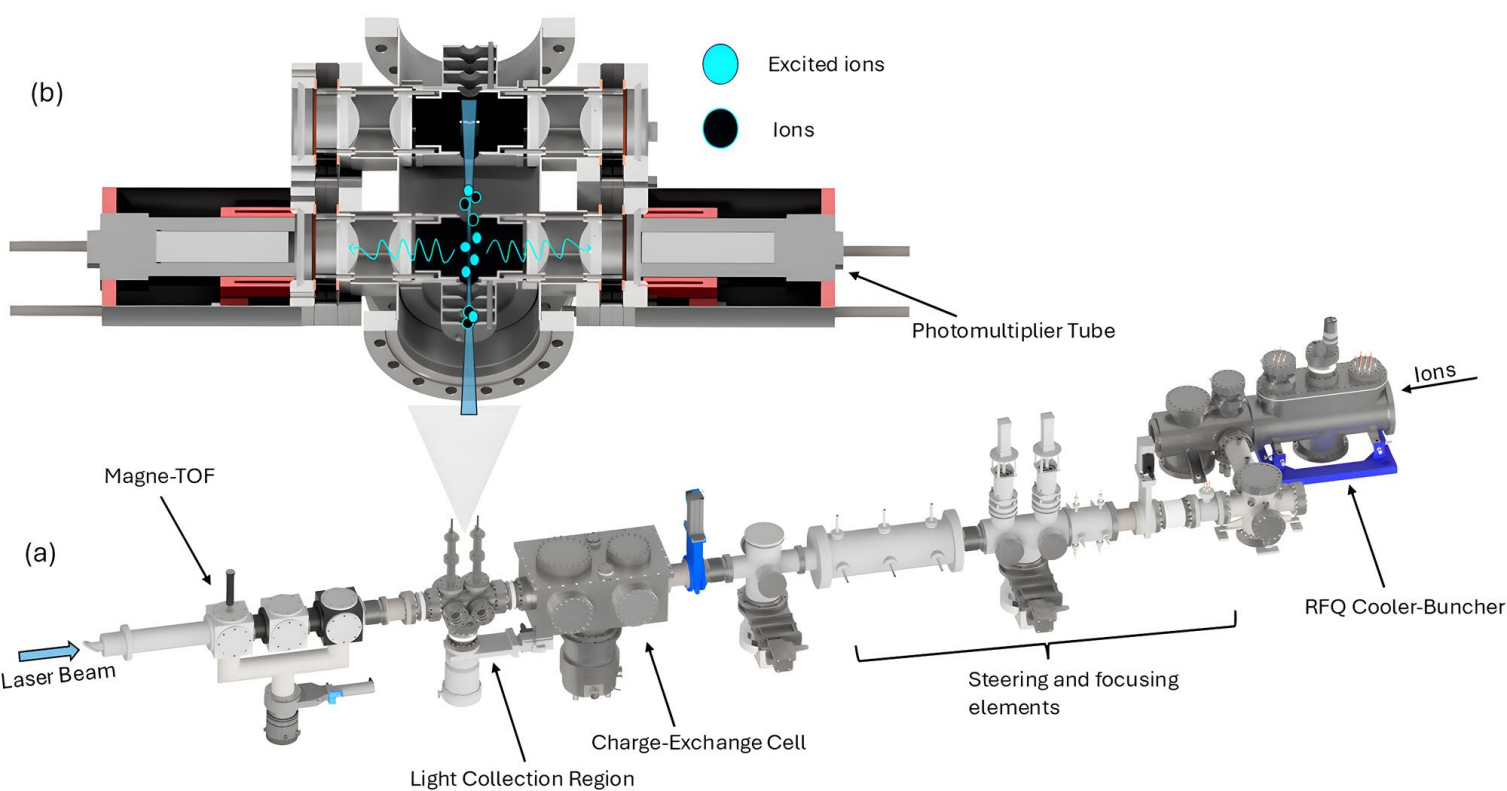
Schematic representation of the collinear laser spectroscopy beamline used for the study of $^{50,51}$V$^{+}$ at IGISOL. **(a)** Overview of the beamline layout from the RFQ cooler–buncher to the light-collection region (LCR). Ion bunches extracted from the RFQ are steered and focused onto the collinear beamline axis and delivered to the LCR. Resonance fluorescence is detected by two photomultiplier tubes (PMTs), while a Magne-ToF detector downstream is used for ion-beam diagnostics. A charge-exchange cell located upstream of the LCR enables neutral-atom spectroscopy when required. **(b)**Cross-sectional view of the LCR shown as viewed from above, with the viewing direction perpendicular to panel (a) and parallel to the ion and laser beam axes. The anti-collinear overlap between the ion beam and the continuous-wave laser beam is illustrated. Excited ions emit fluorescence photons, which are collected by the aspheric lenses and directed onto the PMTs. Scheme in (a) is adapted from Ref.^26^.

In the present work, the CLS technique was implemented at the IGISOL IV facility of the JYFL Accelerator Laboratory, University of Jyväskylä, Finland^22,23^. Singly charged vanadium ions, $^{50,51}\textrm{V}^+$, were produced in a discharge ion source operated with a helium buffer gas at a pressure of approximately 25 mbar. The ions extracted from the source are electrostatically guided to the high-vacuum region of the beamline via a radiofrequency sextupole ion guide and a differential pumping stage^24^. The extracted ion beam was accelerated to an energy of 30 keV and mass-separated using a dipole magnet with a resolving power of $m/\Delta m \approx 300$, sufficient to create beams of $^{50}V$ and $^{51}V$ free from contamination of the other stable isotope. The ions were subsequently injected into a gas-filled radiofrequency quadrupole (RFQ) cooler-buncher^25^. After being accumulated in a helium environment of $\sim 10^{-2}$ mbar, the ions were released as bunches with a temporal width of $\sim 10\,\mu$s at a repetition rate of 10 Hz and transported to the collinear laser spectroscopy line. A schematic overview of the CLS beamline, including the RFQ, steering section and the light collection region, is shown in Fig. 1.

The continuous-wave laser light used to probe the vanadium ionic transitions was generated using a HÜBNER Photonics C-WAVE GTR tunable laser system. In this work, the internally generated second-harmonic output (2$\omega$) of the C-WAVE GTR was actively stabilized to the desired wavelength by locking this beam to a HighFinesse WSU-10 wavelength meter. The 2$\omega$ beam was then mode-matched in free space into an external Sirah WaveTrain frequency-doubling cavity, where an additional second-harmonic generation stage provided fourth-harmonic ultraviolet light (4$\omega$) in the wavelength range of 288–312 nm. The laser power used was in the range of 1–2.5 mW depending on the transition strength, and was optimized through saturation measurements to minimize power broadening in the spectra.

The ions were overlapped with the laser beam in an anti-collinear geometry inside the light-collection region (LCR). The LCR consists of a 34 cm-long vacuum chamber equipped with four optical collection ports, of which the first two were used in the present work. A vacuum level of $10^{-7}$ mbar was maintained in the LCR to suppress background coming from collisional excitations. The use of bunched beams enables a substantial reduction of background signals by applying time-of-flight gating to the photon detection of the laser-induced fluorescence corresponding to the arrival time of the ion bunch at the interaction region. The fluorescence photons were collected by two Asphericon AFL50-60-S-U aspheric lenses (50 mm diameter, 60 mm focal length) and detected by two H11123 Hamamatsu photomultipliers (PMTs) placed on electrically insulated mounts on the outside of the vacuum chamber viewports. During beam tuning, a 1 mm retractable aperture was used to optimize the spatial overlap between the ion and laser beams. More information about the design of the LCR and other updates on the CLS beamline can be found in Refs^27,28^.

Doppler tuning of the ionic resonance was achieved by varying the electrical potential of the entire LCR. The voltage applied to the chamber was controlled using a 16-bit Measurement Computing USB-3102 digital-to-analog converter, whose output (±10 V) was amplified by a $\times$1000 TREK 609E-6 high-voltage amplifier. Prior to every measurement, a calibration of the voltage-to-frequency conversion was performed. The LCR voltage and the RFQ platform potential were monitored with two Keysight 34465 A digital multimeters, using 1:1000 and 1:10000 voltage dividers, respectively. The PMT signals were recorded event-by-event using a Cronologic TimeTagger 4-2G time-to-digital converter, providing 500 ps timing resolution. For each measurement, all relevant experimental parameters such as laser frequency setpoint, RFQ platform potential, LCR voltage settings, and the time-stamped photon events were recorded in a single data file, ensuring a complete and synchronized dataset for the subsequent analysis.

A total of 11 transitions in singly ionized vanadium were investigated, covering the wavelength range of 288–312 nm. All transitions correspond to the $3d^3(^{4}F)4s \rightarrow 3d^3(^{4}F)4p$ configuration. The fact that the ions were cooled to near room-temperature combined with the 30 keV acceleration, resulted in narrow spectral linewidths. Hyperfine structure measurements were performed for $^{51}\textrm{V}$, allowing extraction of the magnetic dipole (*A*) and electric quadrupole (*B*) hyperfine parameters. In addition, isotope shift measurements were conducted for $^{50}\textrm{V}$ relative to $^{51}\textrm{V}$ for 5 of the 11 studied transitions.

## Data analysis

Since the ions were accelerated to 30 keV, the laser frequencies measured in the laboratory frame were first converted into the rest frame of the ions. For an anti-collinear geometry, the rest-frame transition frequency $\nu _{0}$ is related to the laboratory-frame laser frequency $\nu _{\textrm{lab}}$ through the relativistic Doppler formula :

1 $$\begin{aligned} \nu _{0} = \nu _{\textrm{lab}} \sqrt{\frac{1+\beta }{1-\beta }}, \end{aligned}$$

where $\beta = v/c$ is the ion velocity in units of the speed of light. The ion velocity was determined from the total applied acceleration voltage *V* using

2 $$\begin{aligned} \beta = \sqrt{1 - \left( \frac{mc^{2}}{mc^{2} + qV} \right) ^{2}}, \end{aligned}$$

with *m* and *q* denoting the ion mass and charge, respectively. For each voltage step in the scan, the exact Doppler transformation given in Eq. (1) was applied to obtain the rest-frame frequencies.

The extraction of the hyperfine structure (hfs) parameters and isotope shifts from the recorded fluorescence spectra was performed through a multi-step analysis procedure. The analysis follows the standard formalism of atomic hyperfine interactions^29^ and is adapted to the experimental conditions of collinear laser spectroscopy. For each transition, the measured resonance profile consists of a set of hyperfine components corresponding to the allowed transitions between the hyperfine levels of the upper and lower electronic states. The positions of these components were used to determine the magnetic dipole (*A*) and electric quadrupole (*B*) hyperfine coupling constants, as well as the hyperfine centroid transition frequency required for the isotope-shift analysis.

The energy shift of a hyperfine level with quantum number *F* is expressed up to second order in terms of the hyperfine constants *A* and *B* as:

3 $$\begin{aligned} E_{F} = \frac{1}{2} A K + B \frac{\frac{3}{4}K(K+1) - I(I+1)J(J+1)}{2I(2I-1)J(2J-1)}, \end{aligned}$$

where *I* is the nuclear spin, *J* is the total electronic angular momentum of the state, and

4 $$\begin{aligned} K = F(F+1) - I(I+1) - J(J+1). \end{aligned}$$

The hyperfine constants are related to the underlying nuclear electromagnetic moments by

5 $$\begin{aligned} A = \frac{\mu B(0)}{I J} \qquad \text {and}\qquad B = e Q_{s} V_{zz}, \end{aligned}$$

where $\mu$ is the nuclear magnetic dipole moment, *B*(0) is the magnetic field produced by the electrons at the nucleus, $Q_s$ is the spectroscopic nuclear electric quadrupole moment, and $V_{zz}$ is the principal component of the electric field gradient at the nucleus. The allowed hyperfine transitions between an upper state $F'$ and a lower state *F* obey the selection rules $\Delta F = 0, \pm 1$, except for $F = 0 \leftrightarrow F' = 0$ which is forbidden. The relative intensities of the hyperfine components were fixed during the fit according to the electric-dipole transition strengths calculated using Racah algebra, expressed in terms of Wigner 6*j* coefficients. The line strength for a transition $F \rightarrow F'$ is given by

6 $$\begin{aligned} S_{F \rightarrow F'} \propto (2F'+1)(2F+1) \begin{Bmatrix} J'&J&1 \\ F&F'&I \end{Bmatrix}^{2}, \end{aligned}$$

ensuring physically meaningful and properly normalised line strength ratios for all transitions.

The spectral analysis was carried out using the satlas2 Python framework^30^. The software generates the hyperfine spectral models based on the atomic and nuclear structure parameters of each transition and isotope, and provides different statistical fitting approaches for the extraction of the line-shape parameters and hyperfine constants. To accurately reproduce the experimental spectra, each hyperfine component was modelled by a Voigt line shape, defined as the convolution of a Lorentzian and a Gaussian profile. The Lorentzian part primarily accounts for the natural linewidth of the transition and any power broadening effects, while the Gaussian contribution represents the residual Doppler width arising from the finite ion temperature and laser bandwidth. For each transition, a common Lorentzian and Gaussian width was imposed for all hyperfine components to maintain consistency with the physical broadening mechanisms. A constant background term was included to account for residual scattered light and detector dark counts. The model line shape for a transition is expressed as

7 $$\begin{aligned} S(\nu ) = \sum _{i} I_{i} \, V\big (\nu - \nu _{i}; \sigma , \Gamma \big ) + B_{0}, \end{aligned}$$

where $I_{i}$ and $\nu _{i}$ are the relative intensity and frequency of the *i*-th hyperfine component, respectively, $\sigma$ and $\Gamma$ denote the Gaussian and Lorentzian contributions to the Voigt profile, and $B_{0}$ is the constant background level. The Voigt function $V(\nu ; \sigma , \Gamma )$ was evaluated numerically for each fit point.

The hyperfine constants were extracted by fitting the experimental spectra using two independent statistical approaches. First, a standard $\chi ^{2}$ minimization was performed, assuming Poissonian counting statistics for the data points, which provided the best-fit parameters and their associated statistical uncertainties. In addition, a Markov Chain Monte Carlo (MCMC) analysis was carried out as an independent verification of the fitted values. The MCMC sampling yielded posterior probability distributions for the model parameters, serving as a consistency check on the results obtained from the $\chi ^{2}$ minimization. Both methods produced mutually consistent parameter values, providing confidence in the robustness of the extracted hyperfine constants across all investigated transitions. The overall quality of the fits was also assessed through the reduced chi-squared values, which were typically found to lie in the range $\chi ^2_\nu \approx 0.8$–3 across the investigated transitions.

**Fig. 2:**
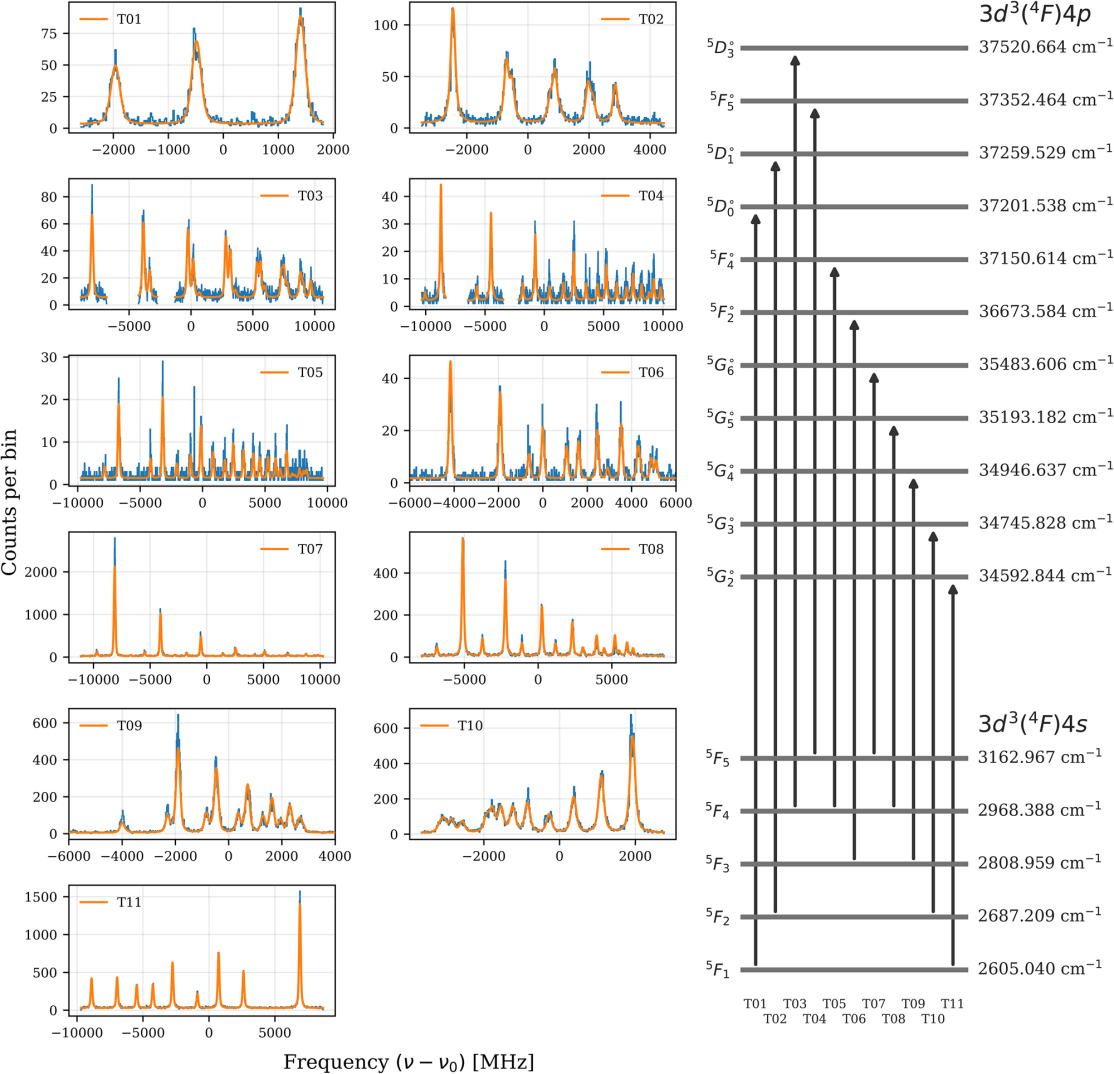
Overview of the hyperfine spectra recorded for the eleven investigated transitions in singly ionised $^{51}\textrm{V}^+$. The left panels (T01–T11) display the experimental collinear laser spectroscopy data (blue) together with the corresponding hyperfine structure fits (orange), plotted as rest-frame frequency detuning $(\nu -\nu _0)$. Each label T01–T11 denotes an internal identifier for a specific transition. The right-hand panel shows the electric-dipole transitions between the $3d^{3}(^4F)4s$ and $3d^{3}(^4F)4p$ fine-structure manifolds, where each level is labelled by its total electronic angular momentum term symbol ($^{5}F_{J}$, $^{5}D_{J}$, $^{5}G_{J}$) and its energy in cm$^{-1}$. The superscript $\circ$ on the upper-level terms indicates odd parity of the 4*p* configuration relative to the even-parity 4*s* states.

The isotope shift between the isotopes *A* and $A'$ for a given transition was then extracted as the difference in centroid frequencies,

8 $$\begin{aligned} \delta \nu ^{A,A'} = \nu _{\textrm{c}}(A) - \nu _{\textrm{c}}(A'), \end{aligned}$$

after converting all fitted frequencies to the rest frame of the ions using Eq. (1). The isotope shifts reported in this work are given with respect to $^{51}$V. Figure 2 shows the fitted hyperfine spectra for all investigated transitions in $^{51}$V$^{+}$ between the $3d^{3}(^{4}F)4s$ and $3d^{3}(^{4}F)4p$ configurations. Each panel corresponds to one of the measured transitions. As expected for vanadium, the combination of the high electronic angular momenta of these states with the nuclear spin $I=7/2$ of $^{51}$V results in a dense hyperfine structure for the majority of the studied transitions.

In order to assess systematic uncertainties in the acceleration voltage, the absolute value of the post–cooler acceleration potential was calibrated using the well-established hyperfine structure of Yb$^{+}$. Because the hyperfine splittings of selected Yb$^{+}$ transitions are known with sub-MHz precision, fitting the Yb spectra provides a direct and highly sensitive measure of any constant offset in the applied acceleration voltage^27,31^. Approximately one month prior to the present vanadium measurements, dedicated calibration runs were performed at the same beamline using the $^{171}$Yb$^{+}$ and $^{173}$Yb$^{+}$ isotopes. The $4f^{14}6s\,\,^{2}S_{1/2} \rightarrow 4f^{14}6p\,\,^{2}P_{3/2}$ transition at 328.937 nm was investigated, and independent fits were carried out while introducing controlled offline offsets to the absolute acceleration voltage. By systematically varying the applied voltage offset and refitting the spectra, the dependence of the extracted ground-state hyperfine magnetic dipole constant *A* on the absolute voltage scale was evaluated. A common offset region for which the extracted *A* values were consistent with the literature values within uncertainties was identified and adopted as the calibrated voltage reference.

## Results and discussion

### Hyperfine parameters of $^{51}$V$^{+}$

Table 1 lists the extracted magnetic dipole (*A*) and electric quadrupole (*B*) hyperfine coupling constants for each energy level of $^{51}$V$^{+}$ analyzed. The level energies *E* are given relative to the ground state, and the quoted uncertainties correspond to one standard deviation. The last two columns provide the normalized values $A/\mu$ and *B*/*Q*, expressed per nuclear magnetic dipole and quadrupole moment expessed in terms of nuclear magneton and barns, respectively.

**Table 1:** Extracted hyperfine coupling constants for the observed energy levels of $^{51}$V$^{+}$. The table summarizes the measured magnetic dipole (*A*) and electric quadrupole (*B*) constants for each level, together with the corresponding normalized quantities $A/\mu$ and *B*/*Q*. For comparison, previously reported literature values ($A_{\text {Lit}}$) are included from Ref.^6^. The level energies *E* and total angular momenta *J* are also listed for reference. Statistical errors are mentioned inside a parenthesis and systematic errors in square brackets.

$E\,(\textrm{cm}^{-1})$	*J*	$A_{Lit}\,(\textrm{MHz})$	$A\,(\textrm{MHz})$	$B\,(\textrm{MHz})$	$A/\mu \,(\textrm{MHz}/\mu _{N})$	$B/Q\,(\textrm{MHz}/\textrm{b})$
2605.04	1	−411(30)	−421.57(36)[30]	10.7(14)[2]	−82.15(8)	−206(45)
2687.209	2	405(90)	354.8(8)[2]	−81(17)[10]	69.14(16)	1558(443)
2808.959	3	552(30)	546.3(6)[1]	−34.2(47)[4]	106.46(13)	658(155)
2968.388	4	615(30)	620.97(57)[80]	−12.2(15)[20]	121.01(12)	235(54)
3162.967	5	654(30)	661.65(68)[40]	−69(21)[5]	128.94(15)	1327(258)
34592.844	2	780(45)	776.55(21)[35]	12.5(21)[3]	151.33(8)	−240(61)
34745.828	3	432(45)	424.46(96)[15]	−57(19)[5]	82.72(19)	1096(214)
34946.637	4	270(45)	281.97(38)[5]	−32.6(41)[7]	54.95(8)	627(144)
35193.182	5	201(45)	208.71(64)[50]	−18.4(34)[25]	40.67(13)	354(94)
35483.606	6	156(60)	166.16(58)[8]	−52(5)[2]	32.38(11)	1000(193)
36673.584	2	–	238.9(12)[2]	−21(7)[1]	46.56(23)	404(79)
37150.614	4	147(45)	149.41(94)[45]	–	29.12(18)	–
37259.529	1	–	42.6(17)[2]	–	8.30(33)	–
37352.464	5	–	159.43(97)[24]	–	31.07(19)	–
37520.664	3	75(45)	75.5(16)[1]	–	14.71(31)	–

The observed resonance spectra exhibited full-width-at-half-maximum (FWHM) values between 100 and 150 MHz, primarily determined by Doppler broadening from the velocity distribution of the ion bunch after the extraction from the cooler. Also in cases of low spectroscopic efficiency, higher laser intensities were required to compensate for reduced signal levels. Under these conditions, power broadening can become significant and contributes to a widening of the observed spectral features. Each transition was measured multiple times under identical conditions, and the final hyperfine parameters reported in Table 1 were obtained as weighted averages of the individual fits. For each dataset, the hyperfine constants *A* and *B* for both the lower and upper electronic levels, as well as the centroid frequency of the hyperfine multiplet, were determined. When a level was accessed by multiple transitions, the hyperfine constants obtained from the separate fits agreed within one standard deviation, and the reported values (and uncertainties) represent their weighted averages.

In total, five independent parameters were extracted from the analysis of each transition. Given that the hyperfine spectra typically contain more than five resolved components, the corresponding system of equations used in the least-squares fitting is overdetermined. This condition minimizes correlations between the fitted parameters and improves the overall robustness of the extracted values. The only exception is transition T01, for which only three hyperfine components were observed. However, the upper level of this transition has $J = 0$ and therefore exhibits no hyperfine splitting, ensuring that the remaining three peaks were sufficient to constrain the fit. The statistical uncertainties on the hyperfine parameters were derived from the covariance matrices of the least-squares fits and cross-checked using a Markov Chain Monte Carlo (MCMC) approach implemented within the Satlas2 framework. The two methods showed excellent agreement, with the MCMC analysis confirming the reliability of the $\chi ^{2}$-based uncertainties, particularly for spectra with lower signal-to-background ratios.

As previously mentioned, the calibration against Yb$^{+}$ fixes the absolute acceleration voltage scale; however, the residual uncertainty of this calibration is on the order of $\sim 10$ V. To account for this, an additional offline voltage–sensitivity analysis was performed: the post–cooler acceleration voltage was artificially varied by $\pm 10$ V around its nominal value in steps of 1 V, and the vanadium spectra were refitted for each setting. This procedure quantifies the sensitivity of the extracted hyperfine parameters to the remaining uncertainty in the acceleration potential, ensuring that any possible systematic shift arising from voltage fluctuations is properly evaluated. The resulting spread in the fitted hyperfine parameters was taken as the estimate of the systematic uncertainty, and is quoted as the second uncertainty (in square brackets) in Table 1.

A direct comparison between the present results and the literature values^6^ reveals a substantial improvement in precision. The hyperfine magnetic dipole constants (*A*) obtained in this work are determined with uncertainties typically two orders of magnitude smaller than those reported previously. In addition, the present study provides the first experimental determination of the electric quadrupole coupling constants (*B*) for $^{51}$V$^{+}$. The statistical uncertainties associated with the *B* constants are systematically larger than those of the *A* constants. This behaviour is expected, as the quadrupole interaction is intrinsically a second–order effect in the hyperfine Hamiltonian, and therefore induces much smaller shifts in the spectrum for comparable coupling strengths. Consequently, unless the *B* values are unusually large, the spectroscopic sensitivity to the quadrupole contribution is reduced, leading to weaker constraints on *B*. For four of the excited levels, the extracted quadrupole contribution was found to be negligible within the experimental sensitivity, resulting in large relative uncertainties. In these cases, no meaningful constraint on *B* could be obtained and the corresponding values are therefore not quoted in Table 1. Despite this, the extracted *B* values for the remaining levels represent valuable new benchmarks for atomic–structure calculations. The hyperfine field coefficients were extracted using as reference the nuclear magnetic dipole moment $\mu =+5.1315(24)\mu _N$ and electric quadrupole moment $Q=-0.052(10)b$from Refs^32,33^., obtained from high–precision nuclear magnetic resonance studies.

### Isotope shifts

**Fig. 3:**
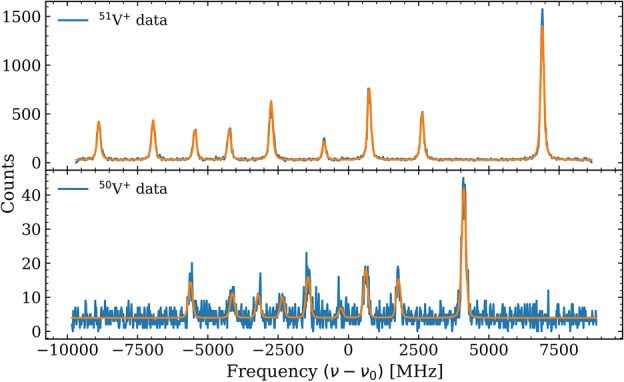
Comparison of the fitted fluorescence spectra for the T11 transition in $^{51}$V$^{+}$ (top) and $^{50}$V$^{+}$ (bottom), as a function of rest-frame frequency detuning $(\nu -\nu _0)$.

Isotope shift measurements were performed for five of the investigated transitions (T07–T11), for which spectra of both $^{50}$V$^{+}$ and $^{51}$V$^{+}$ could be obtained. These transitions correspond to those with the highest spectroscopic efficiency, where the signal-to-background ratio remained sufficient to resolve the hyperfine structure even for the less abundant isotope $^{50}$V (natural abundance $\approx 0.25\%$). The good fluorescence yield and stability of these transitions enabled reliable fitting of both isotopes under identical experimental conditions. As an example, Fig. 3 presents the fitted spectra for the T11 transition in $^{50}$V$^{+}$ and $^{51}$V$^{+}$.

**Table 2:** Measured isotope shifts $\delta \nu ^{50,51} = \nu _{50} - \nu _{51}$ for the five investigated transitions in singly ionized vanadium. The transition ID refers to the internal labeling used throughout this work, and $\lambda$ denotes the corresponding rest-frame transition wavelength. Once again statistical uncertainties are mentioned in the parenthesis and systematic uncertainties in square brackets. All wavelengths refer to vacuum values.

Transition ID	Transition wavelength $\lambda$ (nm)	$\delta \nu ^{50,51}$ (MHz)
T07	309.310	−404(10)[2]
T08	310.230	−418(7)[4]
T09	311.071	−383(6)[3]
T10	311.838	−426(5)[1]
T11	312.529	−414(3)[2]

For each transition, the centroid positions of the hyperfine multiplets for both isotopes were precisely determined from the hyperfine fits. Since the two isotopes were measured consecutively and with the same laser frequency setpoint, the isotope shifts were determined in a strictly differential manner. In this approach, any constant offset in the absolute laser frequency or in the Doppler-tuning voltage cancels in the difference between the two isotopes. While small drifts of the laser frequency or ion-beam acceleration voltage between successive scans cannot be excluded in principle, repeated reference scans confirmed that both quantities remained stable on the timescale of the measurements. Consequently, both isotopes were effectively probed under the same experimental conditions, allowing a reliable extraction of the relative isotope shifts. The extracted shifts for all five measured transitions are summarized in Table 2.

## Conclusions

High-resolution collinear laser spectroscopy was performed on singly charged vanadium ions at the IGISOL facility, enabling precise determination of the hyperfine structure and isotope shifts of $^{50,51}$V$^{+}$. Eleven transitions between the $3d^{3}(^{4}F)4s$ and $3d^{3}(^{4}F)4p$ configurations were investigated, yielding magnetic dipole (*A*) and electric quadrupole (*B*) coupling constants with substantially improved precision. The present measurements improve the precision of existing *A* values up to nearly two orders of magnitude and report *B* constants for the first time in vanadium ions. For five of the strongest transitions, isotope shifts between $^{50}$V and $^{51}$V were also determined, providing a first experimental benchmark for future studies of field and mass shift contributions along the vanadium isotopic chain. These data will support refined atomic-structure calculations and contribute to the extraction of nuclear mean-square charge-radius differences.

An important outcome of this work is the identification of the transitions with the highest spectroscopic efficiency, defined as the detected fluorescence photons per number of incoming ions. In particular, transitions T07–T11 showed the best performance while retaining clear sensitivity to both hyperfine coupling constants and isotope shifts. These transitions therefore constitute prime candidates for future online measurements of radioactive vanadium isotopes. IGISOL is capable of delivering isotopes in the mass range A = 46–52 with ion yields expected to be suitable for collinear laser spectroscopy. The present results thus establish the experimental basis for extending high-precision laser spectroscopy to exotic vanadium isotopes and for systematic investigations of nuclear moments and charge radii in the *fp*-shell region.

## Data Availability

The data supporting this study, together with additional notes on the analysis, are available in the Zenodo repository: https://doi.org/10.5281/zenodo.17910031. Further questions regarding the data or analysis can be addressed to the corresponding author.
